# Understanding Experiences of Youth with Long COVID: A Qualitative Approach

**DOI:** 10.3390/children11030335

**Published:** 2024-03-12

**Authors:** Chelsea Torres, Kensei Maeda, Madeline Johnson, Leonard A. Jason

**Affiliations:** Center for Community Research, DePaul University, Chicago, IL 60614, USA; ctorre45@depaul.edu (C.T.);

**Keywords:** Long COVID, youth, qualitative, healthcare

## Abstract

There is limited information on the specific impacts of Long COVID in youth. Long COVID presents as persisting or new symptoms following initial COVID-19 infection. The aim of this study was to better understand how children and their families describe their experiences seeking diagnosis and support following the onset of symptoms of Long COVID. Six children and five caregivers located in the United States participated in this study. Study procedures included an online video interview with caregiver–child dyads. Interview transcriptions were then analyzed using a conventional approach to content analysis, with two independent coders generating themes. Eight themes emerged from this analysis including the severity of illness and symptomatology, difficulty surrounding the diagnostic process and not being believed, the impact on family and social connections, poor school functioning, positive coping, subsequent positive medical experiences, mental health, and knowledge of the medical field and healthcare experience. Themes revealed difficulty for youth and families in navigating the medical system and functioning in areas of daily life as well as areas of positive experiences related to coping and medical involvement. These findings also highlighted areas of needed improvement for the medical community and for research on Long COVID in youth.

## 1. Understanding Experiences of Youth with Long COVID: A Qualitative Approach

As of March 2023, over 676 million worldwide cases and 103 million cases of SARS-CoV-2 virus infection in the U.S. have been reported [1]. In addition to direct infections, there have been medical, psychosocial, and economic hardships as a result of the COVID-19 pandemic [2,3,4]. Vaccination efforts to reduce the spread and severity of the virus have been highly effective, with over 230 million Americans fully vaccinated [5]. Subsequent variants of the virus have been shown to be less severe, alleviating many fears and tensions about the pandemic [6].

Much focus has been on addressing the direct illness and spread, but as the pandemic winds down, there are renewed efforts toward understanding the long-term impacts of SARS-CoV-2 viral infection [7]. Long COVID, also known as Post-Acute Sequelae of COVID-19 (PASC), is a condition in which people infected with COVID-19 experience lingering symptoms for weeks or months after they recover from the initial illness [8]. Due to its recency and the active interest surrounding it, there are many Long COVID case definitions that disagree on its symptomatology and prognosis [9]. There are a wide range of symptoms attributed to Long COVID, but the most common symptoms are fatigue, chest and throat pain, abnormal breathing, and anxiety or depression [10]. Currently, diagnosis of Long COVID is predominantly based on prior infection of SARS-CoV-2 and exclusion of other illnesses that can explain symptoms [11]. Meta-analysis studies reveal that between 31–67% of people with COVID-19 will go on to develop Long COVID past six months based on nine studies [12]. While the underlying biological mechanisms are still unknown, there are several theories currently being investigated [13].

The pediatric population is frequently overlooked when it comes to COVID-19. Youth were far less likely to suffer severe reactions to COVID-19 and experience hospitalizations and death [14]. Approximately 74.9% of children do not have typical COVID-19 symptoms, and those experiencing severe COVID-19 symptoms are more likely to have comorbidities [15,16]. However, a low severity rate does not mean that Long COVID is nonexistent or not impactful in children. Research is scarce and often conflicting, but there are estimates that between 4–66% of children with COVID-19 develop long-haul symptoms [17,18]. This wide variance in prevalence rates and general understanding of Long COVID is due to several factors, including lack of clear definitions in children, similar to adults, differing timelines for follow up in studies, and inconsistent use of control groups [19]. There is evidence to suggest that pediatric Long COVID symptomatology may be similar to adult Long COVID [20,21].

Despite the growing evidence that Long COVID is present in children, there are few studies on the impacts of the illness on children. One study found that children with confirmed COVID-19 had significantly lower quality of life measurements than controls after four months [22]. Another study found several symptoms continued to be reported at higher levels following acute COVID-19 in youth, including but not limited to, fatigue, difficulty falling asleep, neurocognitive symptoms, dead/heavy feeling after exercise, rashes, allergies, pain, and hair loss [23]. Another study sought to explore risk factors for youth developing Long COVID, with factors identified including severity of initial illness, recurrent COVID-19 infections, and hospitalization during acute illness [24]. It has been noted with other pediatric chronic illnesses that children are likely to have less academic achievement and psychosocial development than children without chronic illnesses [25]. Chronic illness also places heavy demands on the family members of children; however, family relations can also serve as a protective factor against further negative impacts [26].

An initial quantitative study by these authors revealed the presence of some persisting symptoms following initial COVID-19 infection, including fatigue and neurocognitive symptoms, among a sample of youth [23]. These findings sparked additional questions related to the individual experiences of youth with Long COVID given the long-term symptoms reported. By understanding the specific experiences of children with Long COVID and the various impacts the illness has on their lives, better support structures and advocacy can be levied in their favor. Therefore, the current study aims to explore the unique lived experiences of pediatric Long COVID participants and their families who are coping with the implications of their ongoing illness utilizing a qualitative approach in efforts to amplify patient voices and better identify what is important to families related to investigating Long COVID.

## 2. Method

### 2.1. Participants

Participants included six caregiver–child dyads that were composed of six children and five caregivers. All participants were located in the United States. Caregivers interviewed in tandem with each child who had previously been ill with COVID-19. One caregiver participated with two of her children, and therefore was interviewed with each child separately. Interviews were conducted in March and April of 2021. To meet study inclusion criteria, children and caregivers were required to speak and understand English, children were between the ages of 5–17 years old, and caregivers were 18 years or older. Additionally, children were required to have experienced long-term symptoms after becoming ill with COVID-19. As little information about Long COVID in youth was available at the time of recruitment, a wide selection criterion related to age was selected in efforts to best understand the illness across childhood.

The study resulted in a total sample size of 6 youth (66.7% female) with a mean age of 13.6 years (*SD* = 3.98, Range: 7–17). Five children identified as White and 1 child identified as Asian. Participants were asked to provide an approximate date of initial COVID-19 onset, with a mean duration of 40.5 weeks (*SD* = 15.9) since initial onset across participants. All caregivers who participated in this study were mothers.

### 2.2. Procedure

Participants were recruited via convenience sampling through various COVID-19 patient blogs, myalgic encephalomyelitis/chronic fatigue syndrome (ME/CFS) web-based organizations, and social media platforms. Recruitment messages contained information regarding study procedures and goals, such as the intent to better understand the unique experiences of youth who were struggling with ongoing illness following COVID-19 infection. Recruitment postings included a link to a brief online survey through REDCap [27] to screen for eligibility criteria and receive preferred contact methods from participants. Participants who met criteria were contacted to schedule an online video interview hosted through Zoom which was securely recorded. All participants provided online and verbal consent prior to participating in study procedures, which were approved by the authors’ Institutional Review Board (IRB). The caregiver–child dyads were recorded as a single case and transcribed by a research assistant to be qualitatively analyzed.

### 2.3. Measures

A semi-structured interview method was utilized to enable free responses while ensuring consistency. The initial questions were to gather demographic information, including age, race, and gender. The structured questions opened with, “What symptoms are you currently experiencing? How have symptoms changed since you first became ill?” This was followed by questions aimed at the experiences of participants. These questions were, in order, “Have you felt blamed or stigmatized in any way because of having COVID-19?”; “Have you found ways to engage socially during quarantine/physical illness?”; “How were your interactions with the healthcare system following symptoms of COVID-19?”; “Were you able to get tested? If so, what was the process like?”; “How has COVID-19 impacted your family and close relationships?”; “How has COVID-19 impacted your mental health?”; and “How have your symptoms impacted your ability to do schoolwork or engage with other students?” Prompts to elicit more detailed descriptions were used in addition to the natural flow of conversation.

### 2.4. Analytic Plan

Due to limited understanding regarding pediatric Long COVID, a conventional content analysis approach was utilized [28]. This method of qualitative analysis allows for themes to be derived directly from the data within this study without the use of pre-existing theory or understanding and utilized a manifest approach, focusing on the surface level ideas and experiences described by participants. Organization and analysis of the transcriptions were done using NVivo (Version 20; released in March 2020). Two independent coders first reviewed the transcripts multiple times and identified initial codes. Coders then met to discuss potential themes, and a codebook was devised based on emergent themes that both coders agreed upon. Following construction of the codebook, each coder independently coded all transcripts, and interrater reliability was assessed following final coding.

## 3. Results

The qualitative content analysis revealed different themes within participant responses. These themes are (1) severity of illness/symptomatology, (2) difficulty surrounding the diagnostic process/not being believed, (3) impact on family and social connections, (4) poor school functioning, (5) positive coping, (6) subsequent positive medical experiences, (7) mental health, and (8) knowledge of medical field/healthcare experience. An overview of the themes and descriptors is provided in Table 1. The frequency of each theme across all participants is also reported. Frequency refers to how many independent statements made by either the youth or caregiver occurred for each theme. Each theme is discussed in further detail below, with definitions describing initial codes that encompass each theme, including participants’ comments as examples. Inter-rater reliability was calculated using Cohen’s Kappa, with an average Kappa value of 0.90 across all codes.

### 3.1. Severity of Illness/Symptomatology (n = 263)

This theme included codes of both acute and lasting symptoms participants experienced following infection. All participants mentioned experiencing acute COVID symptoms, such as fever, cold symptoms, fatigue, nausea, and loss of sense of smell at the start of their illness, then progressing into one or several of the following: severe skin rashes and/or lesions, narcolepsy, sleep dysfunction, fatigue, medial arcuate ligament syndrome (MALS), brain fog/neurocognitive dysfunction, gastrointestinal problems, pain, and fever. Participants also often described a relapsing and remitting pattern to these symptoms, in which the child would begin to improve and then experience a subsequent increase in symptoms. For example, Caregiver 5, mother of an 11-year-old female, began describing persistent symptoms following infection and the pattern in which they were occurring:

“Um, brain fog. Trouble concentrating, trouble thinking, trouble remembering, um… red eyes, kind of like from the start it’s just been on repeat and it just keeps coming back”.

Child 2, a 16-year-old female, detailed episodes that occurred sporadically:

“Well, I did have these like ‘flares’ is what we’ve been calling them, um, since November, where I will get like a low-grade fever sometimes and like my face gets really red. It really, like my skin feels really hot like I can feel myself kind of radiating heat, but physically I feel really cold, if that makes sense?”

Her mother, Caregiver 2, further elaborated on additional, unusual symptoms that have occurred following infection:

“So she’s had a long, complicated case. So she’s spent three months in the hospital. Her manifestation has been these strange skin lesions. So when these skin flares start she has, her skin starts off red and swollen and then it condenses inward and then the skin opens up and it looks like a burn or blister… so it would look almost like a burn with a curling iron… but she didn’t touch it. And, um, they’re very painful”.

Further, participants described being diagnosed with other illnesses following initial COVID-19 infection, which further complicated their symptom presentation and led to questions regarding whether initial infection had a direct link to subsequent diagnoses. These diagnoses included mast cell activation syndrome (MCAS), postural orthostatic tachycardia syndrome (POTS), medial arcuate ligament syndrome (MALS), and narcolepsy.

### 3.2. Difficulty Surrounding the Diagnostic Process/Not Being Believed (n = 146)

All parent–child dyads discussed feelings related to difficulties with medical professionals, including instances in which they felt they were not believed or they felt professionals attributed their symptoms to a psychological cause even though there were physical symptoms. Complicating factors discussed within this theme included limited availability of COVID-19 testing during the beginning of the pandemic, which led to a lack of physical proof of the illness as well as limited knowledge of COVID-19 in children. Child 2 described feeling she was not believed and was left under constant supervision while at the hospital due to medical staff feeling she was hurting herself. She stated, “the most mentally damaging part was the doctors” when discussing her medical journey with Long COVID.

Caregiver 2 discussed frustration with the medical system due to limited knowledge and help:

“I mean I guess I think that, you know, the medical community just does not know enough about COVID. And so when they try to pretend that they’re experts and they know everything, um, is really frustrating because if they would’ve… It’s really the specialists that are the worst… the dermatologists, the ID [infectious disease] docs. If they would just sit down and take a minute to look at the story instead of making accusations off of one lab test, they’d realize that yes, they can’t explain this, but there are so many things you can’t explain cause of COVID and just because you can’t explain it doesn’t mean it isn’t real”. Following multiple hospitalizations for COVID-19, Child 2 eventually received a diagnosis of Long COVID after multiple positive tests for the virus and persistent symptoms.

Caregiver 3, mother of a 7-year-old male, discussed similar feelings, in which there was a sense of isolation due to their child’s symptoms being out of what doctors conceptualized as commonly occurring post viral symptoms:

“What’s the frustrating part is when we meet with doctors… so we’re from [state] so we’ve met with doctors from Children’s, which is a nationally known, renowned… and they’re saying ‘Oh no we haven’t seen this with other kids.’ And I just really wonder, like, how many people are getting lost in the system with all these little things. Or when they flat out say it couldn’t be from COVID that’s surprising to me because we don’t know enough about COVID to say it couldn’t be from COVID”. At the time of this interview, Child 3 still had not received an official positive test or diagnosis related to his symptoms.

Finally, one family detailed how symptoms were passed off as psychological, which contributed to feelings of not being believed. Caregiver 1, mother of a 17-year-old male, stated, “it was just the challenge of like medical professionals responding to it, and just kind of blowing it off, like these are… we just kept getting told these are like psych issues”. Child 1 was hospitalized in the early part of his illness for encephalopathy, received a positive COVID test, and was seen by multiple specialists prior to being told he had Long COVID.

### 3.3. Impact on Family/Social Connections (n = 67)

All participants discussed aspects of disconnection from close social connections and previously enjoyed activity due to Long COVID symptoms.

Caregiver–Child Dyad 2 detailed the impact of family separation and limiting activities due to being sick:

“It’s very scary I mean she was hospitalized for over 3 months and transferred to [hospital name] to try to get a second opinion, so, our family was, you know, split apart for a while”. The child detailed changes in her social patterns, explaining “now I’m only allowed to see my friends at a distance, with a mask on. So that’s made a big, big impact”.

Caregiver 3 described the impact the pandemic as a whole has had on their family, which has been further exacerbated by Long COVID symptoms:

“The collective experience of going through a pandemic together… it’s just different. It’s… we’ve changed our school pattern, our work pattern, like, who we’re seeing, who we’re not seeing and it does impact you”. This caregiver also discussed how this has impacted her child specifically, noting “when we go out he’s more nervous to play on the playground with other kids there, or, you know to be that young and worried about COVID-19”.

### 3.4. Poor School Functioning (n = 60)

This theme included descriptions from all dyads related to worsening school functioning following ongoing COVID-19 symptoms. Difficulties included trouble keeping up with coursework, having to delay academic progress due to illness, and stress related to school difficulties. Symptoms prohibiting school completion included brain fog/cognitive dysfunction, pain, fever, and ongoing management of other symptoms. Dyads reported having to make difficult decisions of remaining in at-home instruction, securing tutors or additional help, or changing schools due to ongoing symptoms and need for increased accommodations that sometimes were not offered.

Child 2 discussed both the stresses of keeping up with schoolwork and being on track to graduate as well as the support she has received from her teachers. This participant reported she has missed approximately one-years’ worth of coursework for some classes due to illness:

“My teachers have always been really good when I was too sick to do anything for a while there. In February I reached out to my teachers and had meetings with them and tried to just start, you know, full force and um, making up with all of my classes and that definitely was super overwhelming, um, yeah not to be able to think as fast or do things as fast as I used to. So, yeah I definitely have a lot of school to make up and it’s been hard with all the different things I have to do if I’m going to graduate on time”.

Caregiver 4, mother of a 17-year-old female, reported her child is “completely withdrawn” from school due to ongoing symptoms such as fatigue and neurocognitive symptoms. She explained the plan moving forward by saying, “when she is better we will catch her up but at this point we’re gonna, she’s gonna lose the year”.

### 3.5. Positive Coping (n = 55)

All participant dyads discussed instances of positive coping throughout the time since initially becoming infected with COVID-19. This theme encompasses any mention of positive occurrences, including helpful social supports, periods of feeling better or recovery from some symptoms, and supportive family interactions. Child 2 described positive encounters with nurses while at the hospital and also expressed how social contact during hospitalization was impactful, stating “a lot of people came to visit me when I was in the hospital, so that was good”.

One family described attempts to connect with others who may share similar illness experiences, with Caregiver 2 reporting, “she made a new friend who also has long-haul COVID who lives in [location]”. Further, this family discussed hopes to continue to foster positive social support among youth with Long COVID, stating “we maybe want to start some sort of long-haul COVID, kind of, kids group where she can like, hang out more somehow”.

Further, some families expressed hope that further research and understanding will help other youth, with Caregiver 3 explaining:

“I’m glad that there’s people doing research for kids, because I think it’s just so important for doctors to know or, you know, we’re coming up with plans for people to move forward in the future”.

### 3.6. Subsequent Positive Medical Experiences (n = 46)

This theme included descriptions in which the family had improved interactions with the medical system. While all of the families initially described feeling frustrated or not believed based on their child’s symptoms, four of the dyads described having better interactions later on in their medical journey. This shift was theorized by some participants to be due to increased knowledge of COVID-19 as the pandemic evolved or having access to physicians or mental health professionals who believed the experienced illness was present. Caregiver 1 discussed the current state of her child’s medical treatment following previous difficulties, explaining “I do have to say, now we have a really good team”.

Child 2 discussed changes in medical care she experienced following her second admission to the hospital:

“The second time I was admitted at children’s, so I was admitted two times and the doctors completely, like, changed. They tried so many more things, they tried experimental things on me. Like you could tell, this Long COVID research they were mentioning it, they treated me. It was night and day, between the way they treated me”. She went on to talk about the way doctors validated her experiences, stating “The things… the things that they said to me, they were saying, like, ‘I’m not gonna give up until we figure this out or it stops’ and, you know, like telling me how proud they are of me and like all this stuff. It was… like I never had a doctor say anything like that”.

### 3.7. Mental Health (n = 40)

This theme included discussions of mental health among participants. These descriptions were both positive and negative, and participants discussed topics such as involvement in mental health treatment, feelings related to the diagnostic process, and stress or fear. Five out of six caregiver–child dyads mentioned themes related to mental health.

When asked about mental health since the beginning of the pandemic and health difficulties, Child 5, an 11-year-old female, stated her mental health was “not the best”. Her mother, Caregiver 5, elaborated by saying, “I would say we’re seeing a lot of depression… a lot of anxiety that we haven’t seen before”. The family explained additional difficulty with sleep, which further impacted mood symptoms. Child 6, who also was interviewed with Caregiver 5, was reported to have mental health symptoms that occurred following Long COVID onset. Child 6, a 14-year-old female, reported the impact of COVID-19 on her mental health to be “a little bit obviously because like I’m in bed a lot but like I don’t think like terrible”. Caregiver 6 further explained, “we have had some tics that are new, and we’ve seen some mania at times”.

Caregiver 3 discussed mood changes in her son following onset of symptoms. She reported “just, sometimes he just can’t deal with life. He gets very emotional like angry or sad and not normal”. This caregiver went on to explain the initial difficulties and stress when multiple family members in the house were experiencing Long COVID symptoms, stating “in the moment, it was really overwhelming”.

On a positive note, Caregiver–Child Dyad 4 discussed a decrease in mental health symptoms following Long COVID. Child 4 stated, “I don’t have OCD [obsessive compulsive disorder] anymore”. Her mother elaborated by saying she had symptoms related to OCD and “when you get sick and have to take to your bed and your rituals were around cleaning up the kitchen and exercising… doing a certain number of exercises every hour on the hour… well you can’t”.

### 3.8. Knowledge of Medical Field/Healthcare Experience (n = 31)

This theme included discussions in which caregivers discussed their own knowledge of the medical field or how caregivers’ healthcare experience shaped advocacy and treatment efforts for their children. Importantly, all five caregivers reported one of the child’s caregivers was a healthcare worker or had significant healthcare knowledge, providing unique insight into how these caregivers may have navigated barriers to care based on insider knowledge into the medical system.

For example, Caregiver 4 discussed the disbelief many healthcare providers exhibited throughout the process of figuring out what was wrong with their child. She explained how her husband’s experience in healthcare shaped their feelings about how they were being treated:

“I mean we sat there, my husbands like…. And again he’s a physician, he’s a surgeon, he deals with these things all the time and he’s like ‘You can’t go to a psychiatric diagnosis until you’ve ruled out physical”. Child 4 went on to be diagnosed with several other diagnoses post initial COVID infection, including MALS and narcolepsy.

Caregiver 1 provided insight into having to advocate for her son throughout the process of receiving medical help:

“I’m a nursing home administrator by occupation, so I’ve been living, breathing, nothing but COVID, so I know a lot about COVID. I have been completely like persistent and like just continuing to advocate for him”.

Finally, Child 2 spoke about her feelings related to how barriers to being believed or receiving adequate healthcare would have been even more pronounced if her family did not have healthcare experience. She stated, “if my parents, like, spoke Spanish or if they even just didn’t work in healthcare it would’ve gone completely differently”. This further highlights the message of this theme that having knowledge of the healthcare field likely helped families overcome certain barriers to receiving diagnoses or treatment.

## 4. Discussion

Themes generated from this study highlighted the severity of Long COVID symptoms in youth, difficulties navigating the healthcare system and impact on various areas of functioning, and positive experiences of social connection and medical support. It is important to note participants in this study contracted COVID-19 and were interviewed prior to widespread availability of vaccines, which are likely to reduce illness severity and long-term complications of infection [29]. Nonetheless, caregiver–child experiences within this study provided evidence for potential severe and long-term impacts of Long COVID, with symptoms that range widely including rashes, fatigue, brain fog, sleep difficulties, and pain. Further, caregiver–child dyads spent time discussing the impacts Long COVID symptoms had on multiple areas of functioning, including in the school, family, social, and mental health domains. This information illuminates the importance of support for youth across all areas of life following Long COVID onset, instead of merely addressing medical complications. Findings also support the idea that Long COVID symptoms vary greatly, and further attention is needed on less common complications, including rashes and relapsing and remitting patterns of illness.

One of the larger themes generated from this study focused on families feeling unsupported during the diagnostic process or not being believed by medical providers. Families described feeling as if providers felt that symptoms were psychiatric or that they could not have occurred from COVID-19 infection either due to a lack of a positive test or of evidence these symptoms have occurred in youth generally. This disbelief led to more contact with the medical system and delayed diagnosis and treatment, further burdening the healthcare system and families. Many participants described subsequent positive encounters with medical providers as more research and understanding of Long COVID was available and as the pandemic progressed. It is our hope that continued research and understanding will allow for faster and more affirmative diagnostic experiences for youth with Long COVID in the future. Nyblade and colleagues [30] described multiple efforts to reduce stigma in healthcare settings, including strategies to enhance provider knowledge of conditions, involving patients in intervention planning and implementation, and changing hospital and policy structures to better address the unique needs of targeted individuals. These strategies may be a helpful starting point for healthcare professionals who regularly work with youth who may be experiencing symptoms related to Long COVID. This study also found that all children had a caregiver who was a healthcare professional or had increased knowledge of the healthcare system. It is therefore important for future research and intervention to consider how those without significant knowledge of the medical system may be negatively impacted or “lost” within the system.

Themes described from participants in this study, including family and social difficulties, school limitations, impacts on mental health, and feelings of disbelief mirror themes discussed in the literature related to myalgic encephalomyelitis/chronic fatigue syndrome (ME/CFS) [31,32,33]. Research surrounding ME/CFS and now Long COVID have highlighted how disbelief can prevent or delay diagnosis of disease and cause stress on patients. Thus, it is vital for continued research and clinical intervention to be investigated to better support youth with Long COVID symptoms. This study has been followed by a larger study currently in progress, which is investigating biological markers and longer-term follow up of youth with Long COVID and will compare outcomes to youth with ME/CFS.

## 5. Limitations

This study relied on self-reporting of symptoms and treatment by families. Participants were not required to have an official diagnosis of Long COVID. Future studies that incorporate additional objective testing may be beneficial to confirm these findings. Additionally, this study used a small, racially and ethnically homogeneous sample size. However, the ages of youth who participated varied, which captures experiences at different developmental periods but may limit some generalizability of the current findings. Given this research was closer to the beginning of the pandemic and less information about Long COVID in youth was available, convenience sampling was used, which likely impacted sample diversity and led to sampling bias. Further sampling bias is evident in the proportion of caregivers with medical knowledge/experience. This likely has impacted the generalizability of findings, given most families had additional healthcare knowledge that may have increased their desire to participate in research and pursue additional testing and medical appointments. Future research on this topic should include efforts to recruit a more demographically diverse sample to better understand how interactions with the medical system and impacts on daily functioning are impacted across differing identities. Further, this study described experiences mostly related to the beginning of the pandemic, with limited testing, vaccine availability, and understanding of Long COVID. The time period of this study also likely led to a smaller sample size, as fewer families had an understanding of this illness. Future research investigating current impacts related to Long COVID as the pandemic has progressed with larger sample sizes would provide further information on additional areas that can be addressed in diagnostic and treatment processes.

## Figures and Tables

**Table 1:** Summary of themes from qualitative analysis.

Theme		*n*
Severity of illness & Symptomatology	Descriptions of the debilitating nature of symptoms, several of which are outside of those commonly understood	263
Difficulty surrounding the diagnostic process & not being believed	Descriptions of feeling as though they were not believed by medical professionals, or that their illness experiences were psychiatric in nature despite evidence of infection	146
Impact on family & social connections	Comments related to being isolated from their friends/family, unable to enjoy activities of life as they were prior to illness onset	67
Poor school functioning	Descriptions of difficulty completing schoolwork, having to delay course completion, and associated stress as a result of these circumstances	60
Positive coping	Any mention of helpful or positive occurrences during COVID-19 illness	55
Subsequent positive medical experiences	Descriptions of experiences in which the child/family had better interactions with the medical system	46
Mental Health	Mentions of impact of COVID-19 on mental health symptomatology	40
Knowledge of medical field & healthcare experience	Mentions that a family member, often a caregiver, was a healthcare professional or had increased knowledge of the medical field, which directly impacted advocacy efforts and increased understanding of what was happening	31
Kappa	0.9002	

## Data Availability

Data are contained within the article.
